# Supplementary figures and images for: Social inequities and clinical outcomes in young women with cervical cancer: Real-world evidence
Source: PLoS One. 2026 Mar 2;21(3):e0343651. doi: 10.1371/journal.pone.0343651 (PMC12952609; doi:10.1371/journal.pone.0343651)

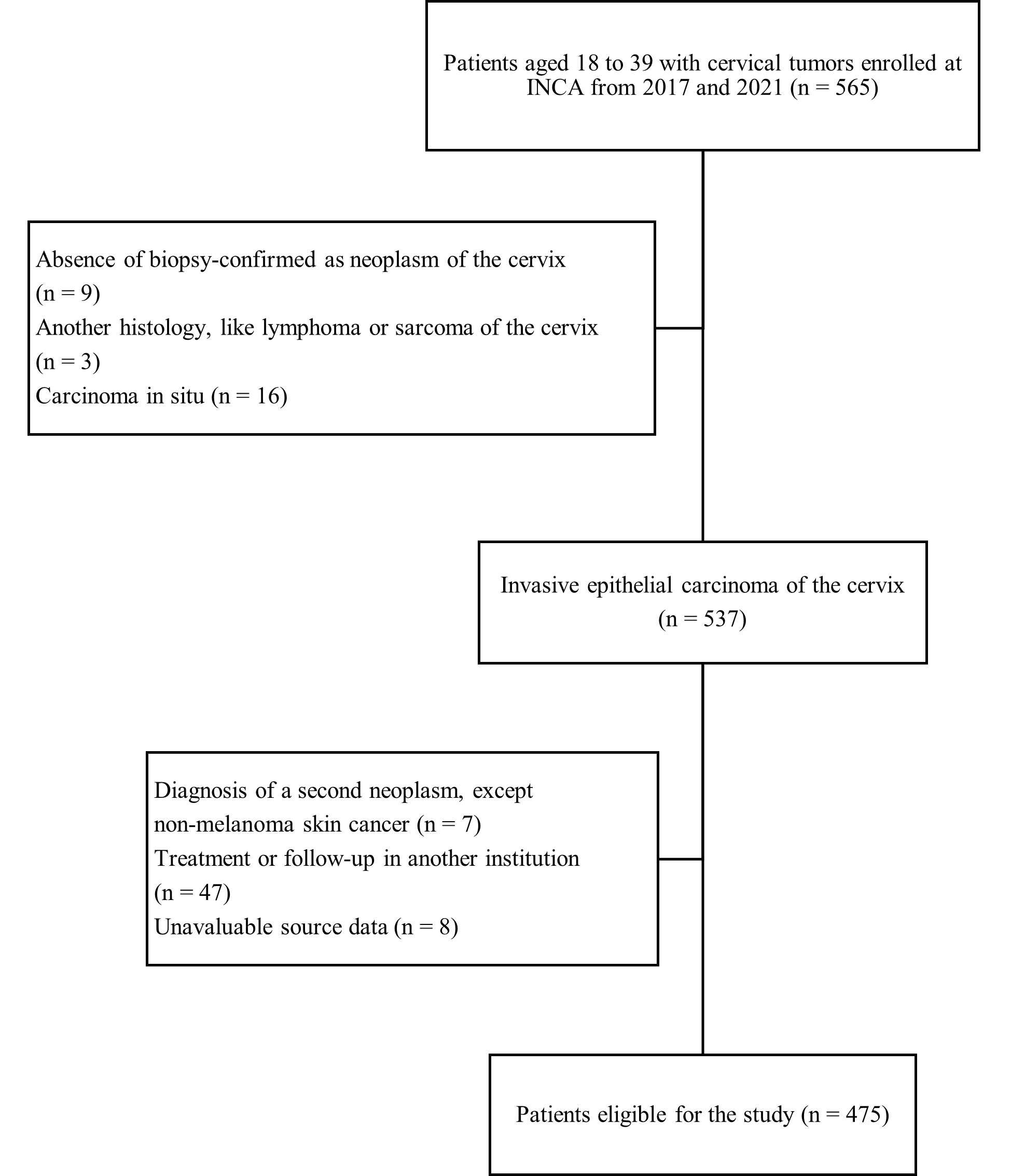

Supplement: S1 Fig — (TIF) [file pone.0343651.s001.tif]

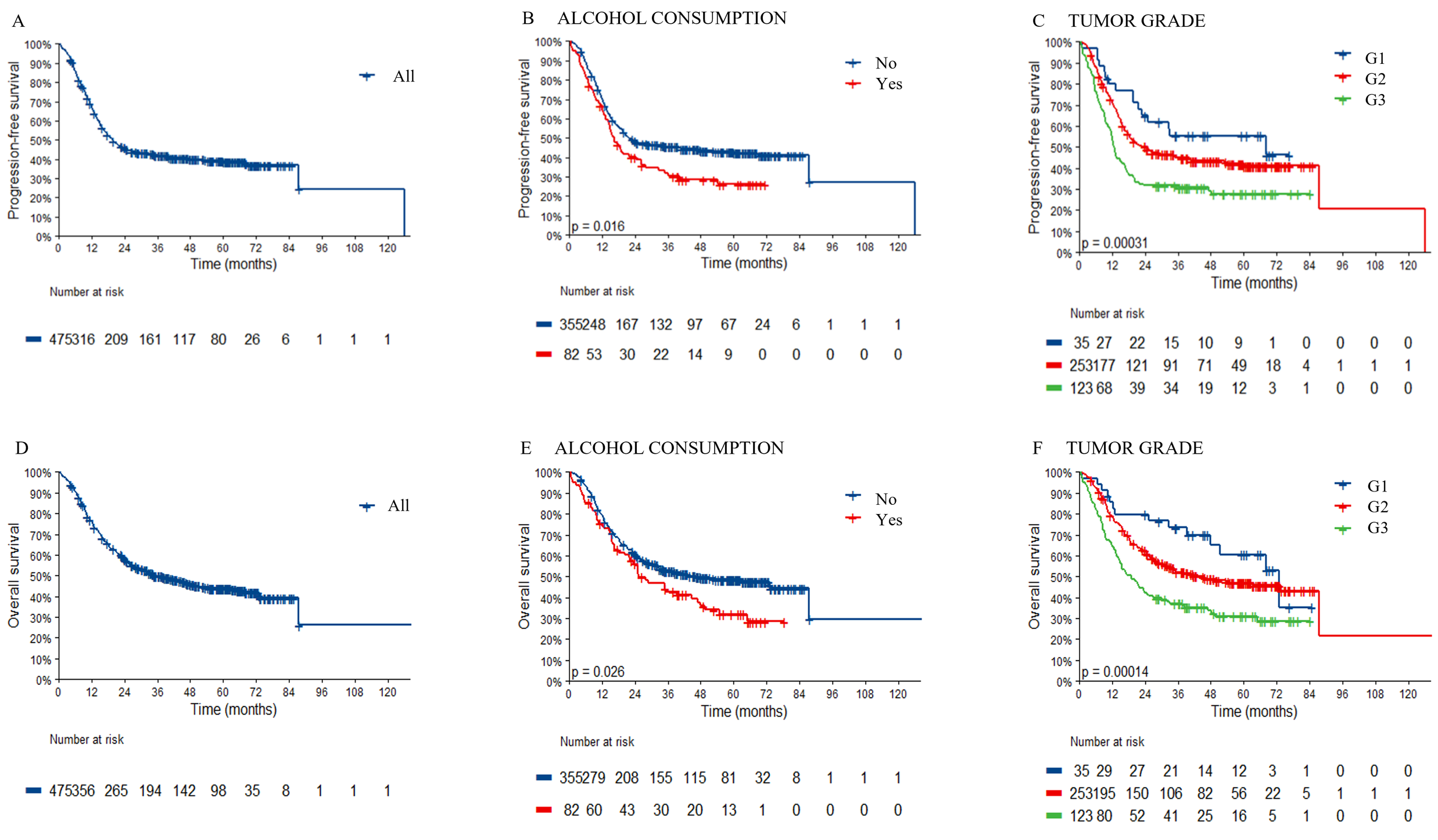

Supplement: S2 Fig — Progression-free survival by A) Overall population, B) Alcohol consumption, C) Tumor grade. Overall survival by D) Overall population, E) Alcohol consumption, F) Tumor grade. Thick marks indicate censored data. (TIF) [file pone.0343651.s002.tif]
