# Supplementary material for: Social inequities and clinical outcomes in young women with cervical cancer: Real-world evidence
Source: PLoS One. 2026 Mar 2;21(3):e0343651. doi: 10.1371/journal.pone.0343651 (PMC12952609; doi:10.1371/journal.pone.0343651)
Supplement: S1 Table — SD, Standard Deviation; BMI, Body Mass Index. (DOCX) [file pone.0343651.s003.docx]

| **Characteristics** | **Total (%)** |
| --- | --- |
| Mean age, years (SD) | 33.6 (4.3) |
| Age < 25 years | 13 (2.7) |
| Age ≥ 25 years | 462 (97.3) |
| Ethnicity | 472 (99.4) |
| White | 162 (34.3) |
| Non-white | 310 (65.7) |
| Marital status | 472 (99.4) |
| Single | 321 (68.0) |
| Married | 126 (26.7) |
| Divorced | 22 (4.7) |
| Widow | 3 (0.6) |
| Education | 468 (98.5) |
| < 8 years | 176 (37.6) |
| ≥ 8 years | 292 (62.4) |
| Mean distance between INCA and habitation, km (SD) | 37.8 (26.5) |
| Smoking | 448 (94.3) |
| No | 338 (75.4) |
| Yes, current smoker | 63 (14.1) |
| Yes, former smoker | 47 (10.5) |
| Mean tobacco load, pack-years (SD) | 13.5 (11.6) |
| Alcohol consumption | 437 (92) |
| No | 355 (81.2) |
| Yes, current consumption | 53 (12.1) |
| Yes, former alcohol consumption | 29 (6.6) |
| Mean BMI (SD) | 26.5 (5.8) |
| Underweight | 23 (5.3) |
| Normal | 173 (40.2) |
| Overweight | 131 (30.5) |
| Obese | 103 (24.0) |
| Comorbidities | 468 |
| No | 389 (83.1) |
| Yes | 79 (16.9) |
| Hypertension | 45 (9.5) |
| Diabetes | 12 (2.5) |
| Chronic Kidney Disease | 2 (0.4) |
| Other comorbidities | 29 (6.1) |
| Sexually Transmitted Infection | 401 (84.4) |
| No | 385 (96.0) |
| Yes | 16 (4.0) |
| Human Immunodeficiency Virus | 13 (4.2) |
| Syphilis | 8 (1.7) |
| Hepatitis B | 2 (0.4) |
| Hepatitis C | 5 (1.1) |
| Other Sexually Transmitted Infection | 1 (0.2) |
| Mean number of sexual partners (SD) | 5.9 (6.0) |
| Mean age at first sexual intercourse, years (SD) | 15.7 (2.2) |
| Mean number of pregnancies (SD) | 2.5 (1.7) |
| Mean number of abortions (SD) | 0.4 (0.7) |
| Histological type | 475 (100.0) |
| Squamous cell carcinoma | 386 (81.3) |
| Adenocarcinoma | 64 (13.5) |
| Adenosquamous | 17 (3.6) |
| Undifferentiated carcinoma | 8 (1.7) |
| Tumor grade | 411 (86.5) |
| Grade 1 | 35 (8.5) |
| Grade 2 | 253 (61.6) |
| Grade 3 | 123 (29.9) |
| Cancer stage | 475 (100.0) |
| I | 136 (28.6) |
| II | 138 (29.1) |
| III | 153 (32.2) |
| IV | 48 (10.1) |
| Metastasis site |  |
| Nodal | 18 (3.8) |
| Lung | 10 (2.1) |
| Bone | 5 (1.1) |
| Peritoneal | 6 (1.3) |
| Liver | 2 (0.4) |
| Adrenal | 0 (0.0) |
| Bowel | 1 (0.2) |
| Other metastasis | 1 (0.2) |
