# Supplementary material for: Social inequities and clinical outcomes in young women with cervical cancer: Real-world evidence
Source: PLoS One. 2026 Mar 2;21(3):e0343651. doi: 10.1371/journal.pone.0343651 (PMC12952609; doi:10.1371/journal.pone.0343651)
Supplement: S2 Table — CRT, Chemoradiotherapy; RT, Radiotherapy; CT, Chemotherapy; CDDP, cisplatin; CP, carboplatin-paclitaxel; IQR, Interquartile Range; PS, Performance Status; BT, Brachytherapy. (DOCX) [file pone.0343651.s004.docx]

| **Treatment / Characteristics** | **I** | **II** | **III** | **IV** |
| --- | --- | --- | --- | --- |
| Number of patients (%) | 136 (28.6) | 138 (29.1) | 153 (32.2) | 48 (10.1) |
| Initial treatment |  |  |  |  |
| Surgery | 87 (64.0) | 2 (1.4) | 3 (2.0) | 0 (0.0) |
| CRT | 45 (33.1) | 123 (89.1) | 94 (61.4) | 8 (16.7) |
| RT | 1 (0.7) | 10 (7.2) | 33 (21.6) | 11 (22.9) |
| CT | 3 (2.2) | 0 (0.0) | 3 (2.0) | 9 (18.8) |
| Exclusive palliative care | 0 (0.0) | 3 (2.2) | 20 (13.1) | 20 (41.7) |
| Surgical resection |  |  |  |  |
| R0 | 80 (92.0) | 1 (50.0) | 1 (50.0) | 0 (0.0) |
| R1 | 5 (5.7) | 0 (0.0) | 1 (50.0) | 0 (0.0) |
| R2 | 2 (2.3) | 1 (50.0) | 0 (0.0) | 0 (0.0) |
| Type of CT |  |  |  |  |
| CDDP | 45 (95.7) | 121 (99.2) | 94 (97.9) | 8 (47.1) |
| CP | 2 (4.3) | 1 (0.8) | 2 (2.1) | 9 (52.9) |
| Median cycles of CT (IQR) | 5.0 (5.0-6.0) | 5.0 (5.0-6.0) | 5.0 (4.0-5.0) | 5.0 (1.0-5.0) |
| CT suspended | 7 (14.6) | 22 (17.9) | 25 (25.8) | 10 (58.8) |
| Worsening PS | 0 (0.0) | 0 (0.0) | 4 (2.6) | 4 (8.3) |
| Infection | 2 (1.5) | 3 (2.2) | 3 (2.0) | 1 (2.1) |
| Kidney dysfunction | 0 (0.0) | 0 (0.0) | 4 (2.6) | 4 (8.3) |
| Refractory anemia | 0 (0.0) | 1 (0.7) | 3 (2.0) | 1 (2.1) |
| Treatment toxicity | 4 (2.9) | 10 (7.2) | 11 (7.2) | 0 (0.0) |
| Socioeconomic issues | 0 (0.0) | 4 (2.9) | 1 (0.7) | 0 (0.0) |
| Allergic reaction to CT | 0 (0.0) | 1 (0.7) | 0 (0.0) | 1 (2.1) |
| Other reasons | 1 (0.7) | 6 (4.3) | 5 (3.3) | 2 (4.2) |
| BT | 49 (37.1) | 109 (82.0) | 86 (66.7) | 5 (27.8) |
| Assessment after treatment performed | 109 (81.3) | 105 (78.9) | 97 (65.5) | 17 (37.8) |
| Response to treatment | 95 (90.5) | 61 (61.6) | 45 (50.0) | 7 (43.8) |
